# Supplementary material for: The Epidemiology of Chickenpox in England, 2016–2022: An Observational Study Using General Practitioner Consultations
Source: Viruses. 2023 Oct 27;15(11):2163. doi: 10.3390/v15112163 (PMC10674747; doi:10.3390/v15112163)
Supplement: Supplementary file 1 [file viruses-15-02163-s001.zip › viruses-2659048-supplementary.pdf]

Table S1. Read and SNOMED codes and descriptions used for varicella.

Code type	Code	Description
Read	A52..	Varicella infection (& [chickenpox])
	A52x.	Varicella with other specified complications
	A52y.	Varicella with unspecified complications NOS
	A52z.	Varicella with no complication NOS
	AyuA2	Varicella with other complications
	AyuA3	Varicella without complications
	F0350	Varicella encephalitis
	X70JE	Perinatal varicella
	XaBsP	Varicella-zoster virus infection
	XaFfz	Varicella virus
	XE0R9	Varicella infection
	Y9994	Varicella meningitis
SNOMED	38907003	Varicella
	23737006	Chickenpox with complication
	240469009	Perinatal varicella
	870310004	Perinatal mucocutaneous infection caused by Human herpesvirus 3
	1132085005	Disseminated perinatal varicella
	3192006	Haemorrhagic varicella pneumonitis
	195911009	Chickenpox pneumonia
	423333008	Exanthem due to varicella
